# Latitudinal Adaptive Strategies of *Tetracentron sinense*: Insights from Functional Traits and Phylogenetic Conservatism

**DOI:** 10.3390/biology15120915

**Published:** 2026-06-11

**Authors:** Luwei Yang, Zheng Yang, Zili Wan, Wenjing He, Hongyan Han, Xiaohong Gan

**Affiliations:** 1 Key Laboratory of Southwest China Wildlife Resources Conservation, Ministry of Education, Nanchong 637009, China; ylw5146@stu.cwnu.edu.cn (L.Y.); 15775821253@163.com (Z.Y.); wzl071@stu.cwnu.edu.cn (Z.W.); hewenjing@stu.cwnu.edu.cn (W.H.); hanhongyan@cwnu.edu.cn (H.H.); 2 College of Life Sciences, China West Normal University, Nanchong 637009, China

**Keywords:** functional traits, phylogenetic constraints, environmental influence, adaptive strategy, *Tetracentron sinense* Oliv

## Abstract

Anthropogenic disturbances and climate warming threaten the rare paleoendemic species *Tetracentron sinense*. To identify the divers of its latitudinal adaptation, we integrated functional trait differentiation, environmental filtering, and phylogenetic conservatism. We measured 35 functional traits (leaf morphology, nutrient stoichiometry, stomatal traits, whole-plant architecture) across four natural populations spanning the species’ latitudinal range: BMXS (Baima Snow Mountain), DFD (Dafengding), FP (Foping), LGS (Leigong Mountain). Using correlation analysis, principal component analysis, and phylogenetic community metrics, we found that *T. sinense* dominated all communities. Populations exhibited divergent strategies: DFD expanded leaf area for light capture under high rainfall and shaded conditions; FP increased height and crown width to compete for light; LGS enhanced nutrient-use efficiency under phosphorus limitation; BMXS promoted phosphorus uptake under nitrogen limitation (N/P < 14). Trait variation correlated significantly with elevation, solar radiation, and temperature. PCA explained 90.44% of total variance, and standardized effect size (SES) values for phylogenetic signals range from −2.031 to 1.973; Phylogenetic signals were stronger in co-occurring taxa than in *T. sinense. T. sinense* populations in BMXS and FP are structured by competitive exclusion, while those in LGS and DFD by habitat filtering. We conclude that *T. sinense* achieves latitudinal adaptation by overcoming phylogenetic niche conservatism through phenotypic plasticity. While leaf economic traits remain evolutionarily conserved and niches in glacial refugium are relatively stable, populations adjust trait syndromes via metabolic shifts and structural trade-offs in response to heterogeneous environmental filters. Identifying these adaptive strategies can guide seed sourcing for restoration efforts under climate change.

## Simple Summary

Human activities and global warming are putting a rare ancient tree species *Tetracentron sinense* at risk. This study aimed to understand how this tree adapts to different climates from south to north. We measured 35 features of the tree, such as leaf size, nutrient content, and overall shape, in four populations living at different latitudes. We found that each population uses a different strategy to survive. For example, one population grows larger leaves to catch more sunlight, while another builds stronger structures to support itself. Others change how they use soil nutrients. These differences are linked to local conditions like elevation, sunshine, and temperature. Interestingly, the tree adapts mainly by changing its physical traits flexibly. Our findings show that *Tetracentron sinense* can adjust to environmental changes in multiple ways. This knowledge helps scientists predict how other ancient plants might respond to climate change and supports efforts to protect endangered species and their habitats.

## 1. Introduction

Accelerated anthropogenic disturbances coupled with climate warming are driving unprecedented biodiversity loss. Rare and endangered species—characterized by narrow niches and constrained adaptability—face escalating extinction risks [1]. As vital proxies for reconstructing paleogeography and ancient floristic evolution, these taxa exhibit exceptional sensitivity to environmental shifts. Consequently, their functional trait dynamics serve as critical indicators for deciphering adaptive mechanisms [2,3]. Plant functional traits integrate survival strategies, resource allocation trade-offs, and stress responses [4]. Although previous studies have demonstrated that climate and soil jointly shape leaf chemical profiles [5], the synergistic effects of environmental gradients, interspecific interactions, and evolutionary constraints on endangered species remain inadequately resolved, especially for paleoendemic taxa experiencing population fragmentation and complex niche competition in heterogeneous sites.

Functional trait differentiation arises from contemporary environmental filtering and deep-rooted phylogenetic legacies, as species optimize fitness via coordinated structural and metabolic adaptations [6,7]. Conventional trait analyses frequently neglect evolutionary histories, leading to overestimations of phenotypic plasticity and misinterpretations of adaptive trade-offs [8]. Phylogenetic metrics (e.g., Blomerg’s K, Moran’s I) can quantify trait conservation and community assembly processes, but their application to endangered relict species—particularly those facing acute niche overlap and competitive displacement—remains limited. While niche metrics (e.g., breadth and overlap indices) can identify competitive hierarchies, they rarely incorporate trait-mediated resource partitioning or phylogenetic assembly [9]. This knowledge gap hinders our mechanistic understanding of how historical contingencies (Pleistocene glaciation and postglacial recolonization) and contemporary selection pressures (current climate and biotic competition) jointly determine the persistence of threatened species across heterogeneous sites.

*Tetracentron sinense* Oliv., the sole extant species of the paleoendemic genus Tetracentron (Trochodendraceae) [10], is a Cenozoic relic dating to the Eocene epoch [11]. As a “living fossil,” it offers critical insights into angiosperm evolution and Tertiary paleofloristic dynamics [12]. In China, this nationally Grade II protected species persists in fragmented populations across southwestern and central mountainous regions, primarily inhabiting ecologically unstable niches (e.g., cliffs and isolated valleys) with limited natural regeneration [13]. Recent research has confirmed that ongoing climate warming significantly reduces the seedling survival rate of *T. sinense*, while the surviving individuals can cope with changing environmental conditions through phenotypic trait plasticity and internal physiological regulation and adaptation [14]. Although previous relevant studies have elucidated its broad niche breadth and horizontal/vertical overlap with sympatric competitors [15,16], significant knowledge gaps persist regarding its adaptive strategies in environmental gradients (e.g., latitude, and microhabitat conditions such as canopy closure and solar radiation). Specifically, the interplay between functional trait plasticity (e.g., along the leaf economics spectrum) and microhabitat heterogeneity remains underexplored across latitudinal and longitudinal gradients. Furthermore, the relative contributions of climatic drivers versus evolutionary constraints (e.g., phylogenetic niche conservatism) to adaptive divergence are unresolved. Crucially, despite documented niche competition risks from sympatric dominants, mechanistic links among trait-mediated resource partitioning, microhabitat filtering, and long-term fitness outcomes lack empirical quantification.

To address these gaps, we propose three mechanistic hypotheses:

(1) If phenotypic plasticity overcomes niche conservatism, functional syndromes will diverge along latitude with southern populations showing narrower reaction norms due to climatic stability.

(2) If evolutionary history constrains adaptive trade-offs in *T. sinense*, traits related to stress tolerance and resource acquisition will display strong phylogenetic signals; weak or non-significant signals indicate plasticity-mediated adaptation.

(3) If competitive exclusion dominates resource-rich communities, phylogenetic overdispersion occurs; under environmental stress, habitat filtering leads to phylogenetic clustering.

To test these hypotheses, we measured 35 functional traits across four natural populations spanning the species’ latitudinal range (26° N–33° N), while quantifying abiotic drivers (microclimate, soil properties) and biotic interactions (community structure, competition). Our integrated framework aims to: (1) clarify whether functional trait syndromes shift predictable with latitude; (2) evaluate whether observed trait variation deviates from predictions of phylogenetic niche conservatism; (3) reveal how community assembly changes across environmental gradients.

## 2. Materials and Methods

### 2.1. Study Area

**Figure 1:**
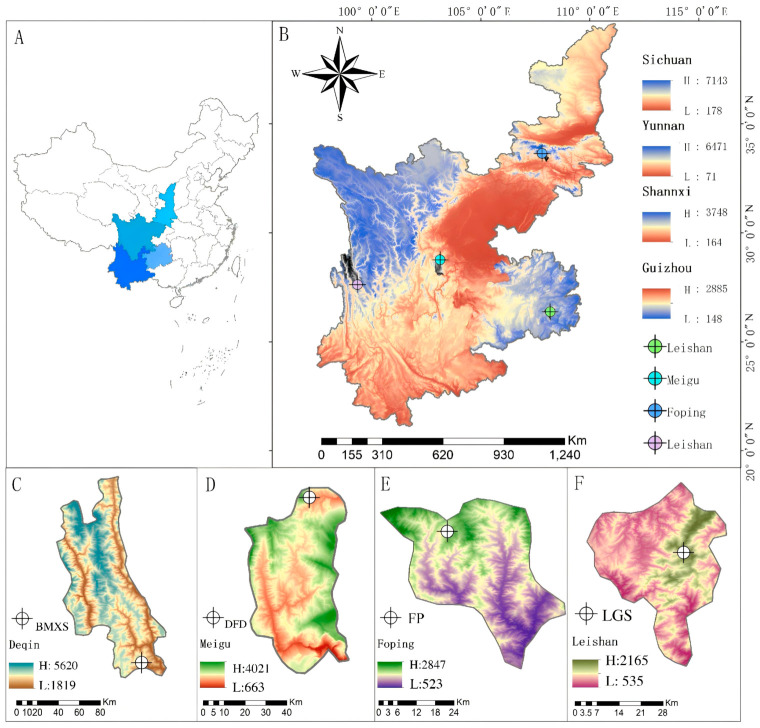
Geographical location of the four study populations of *T. sinense* in China. (**A**) Location of the study area within China (shaded rectangle). (**B**) Topography map of the four provinces (Sichuan, Yunnan, Shaanxi, Guizhou), showing elevation gradients and population positions. (**C**–**F**) Enlarged topographic maps of each population site: (**C**) Deqin County (BMXS population), (**D**) Meigu County (DFD population), (**E**) Foping County (FP population). (**F**) Leishan County (LGS population). In panels (**B**–**F**), the color gradient from green to brown represents elevation from low to high; “H” and “L” markers indicates the highest and lowest elevation value within each mapped area, respectively.

The extant populations of *T. sinense* display a disjunct distribution pattern across East Asian deciduous broadleaf forests, with marked spatial segregation and habitat heterogeneity among metapopulations [17]. We selected four representative populations along latitudinal and historical biogeographic gradients (Figure 1): (1) glacial relicts in the western Hengduan Mountains (Baima Snow Mountain, BMXS: 27°39′ N, 99°21′ E); (2) Last Glacial Maximum (LGM) refugia in southern Sichuan (Dafengding, DFD: 28°46′ N, 103°08′ E); (3) high-latitude populations on the Qinling Mountains’ southern slope (Foping, FP: 33°38′ N, 107°48′ E); and (4) low-latitude populations in southeastern Guizhou (Leigong Mountain, LGS: 26°22′ N, 108°12′ E). These populations collectively exemplify the species’ phylogeographic history, integrating Pleistocene refugial dynamics with contemporary ecological adaptations [18]. Furthermore, they represent distinct evolutionarily significant units and cover a broad latitudinal range, reflecting the species’ distribution.

Site-specific ecological profiles: (1) BMXS (Yunnan Baima Snow Mountain National Reserve) occurs in a subtropical monsoon regime (MAT 8.4 °C; MAP 800–1200 mm) with characteristic associates *Rhododendron decorum*, *Quercus gambleana*, and *Acer flabellatum*. (2) DFD (Sichuan Dafengding National Reserve) inhabits a subtropical humid climate (MAT 9.6 °C; MAP ~1100 mm), co-occurring with relict species *Davidia involucrata* and *Cercidiphyllum japonicum*. (3) FP (Shaanxi Foping National Reserve) represents warm-temperate montane conditions (MAT 11.5 °C; MAP 603.8–1382.3 mm), forming mixed stands with *Dendrobenthamia japonica* and *Cornus controversa*. (4) LGS (Guizhou Leigong Mountain National Reserve), the southernmost population, thrives in subtropical humid climates (MAT 14.3 °C; MAP 1400–1600 mm; >230 frost-free days) alongside *Schima superba* and endemic *Rhododendron leigongshanense*.

### 2.2. Study Site and Plot Establishment

Field surveys were conducted across four distinct *T. sinense* populations (BMXS, DFD, FP, LGS) during June–July 2022 to assess their ecological and functional traits. To ensure representative sampling, three naturally regenerated patches per population were selected based on seed dispersal patterns and geographic heterogeneity. Selected patches exhibited minimal anthropogenic disturbance and maintained a well-structured size distribution. Following the methodology of Weiser [19], a primary sampling plot (100 m ×100 m) was established within each patch, aligned with local topographic gradients (e.g., slope aspect, elevation). To maximize spatial coverage, five standard sampling quadrats (10 m ×10 m) were systematically positioned along diagonal transects within each primary plot, ensuring proportional representation of microhabitats, within a minimum inter-quadrat distance of 25 m. Thus, a total of 60 quadrats (3 patches × 4 populations × 5 quadrats).

### 2.3. Field Sampling

In quadrat, all woody plant individuals with a diameter at breast height (DBH) ≥ 5 cm were georeferenced (latitude, longitude, altitude) using GPS. Topographic variables including slope steepness (°), slope aspect, and concavity index were measured using a digital inclinometer and clinometer. For *T. sinense* and its associated woody species, 8–10 fully expanded, sun-exposed mature leaves were randomly collected from the upper canopy using extendable pruning poles. We measured 284 *T. sinense* individuals (DBH ≥ 5 cm) across populations: BMXS = 80, DFD = 75, FP = 44, LGS = 85. The number of associated species individuals per population is: BMXS = 80, DFD = 77, FP = 49, LGS = 77.

The same field protocol was applied to all co-occurring woody species within each quadrat: individuals with DBH ≥ 5 cm (avoid size-relate allometry) were identified and georeferenced, and 8–10 mature, sun-exposed leaves were collected from three representative individuals per species per quadrat, following the same procedures used for *T. sinense*. This standardized sampling design ensured comparability of functional trait data among species for community-level analyses. All leaf samples were placed immediately in pre-labeled sealed plastic bags with desiccant to reduce water loss and transported to the laboratory under refrigeration (4 °C).

Before trait measurements, leaves were rinsed with deionized water to remove surface contaminants. Leaf fresh mass (LFM) was weighed to the nearest 0.0001 g using an analytical balance. Leaves were then oven-dried at 80 °C for 48 h to constant weight, and leaf dry mass (LDM) was determined. Dried leaf materials were ground into fine powder using a ball mill and sieved through a 100-mesh nylon sieve for subsequent stoichiometric and biochemical analyses.

### 2.4. Functional Trait Quantification

#### 2.4.1. Morphometric Traits

Following Pérez-Harguindeguy et al. [20], the following trait indicators were selected:

Petiole traits: Length (LP), width (PW), length-to-width ratio (LP/PW), perimeter (PP), cross-sectional area (PA).

Leaf blade traits: Blade length (LL), maximum width (LW), length-to-width ratio (LL/LW), perimeter (LC), projected area (LA). 

Leaf and petiole dimensions were analyzed using a dedicated plant image analysis system (LA-S; Hangzhou Wanshen Testing Technology Co., Ltd., Hangzhou, China). Leaf thickness (LT) was measured at three equidistant positions along the midrib using a digital vernier caliper (resolution: 0.01 mm).

#### 2.4.2. Biochemical and Stomatal Traits

Leaf carbon (LCC) and nitrogen (LNC) concentrations were quantified via dry combustion [21] using a VARIO Macro CN elemental analyzer (Elementar, Langenselbold, Germany). Total phosphorus content (LPC) was determined colorimetrically via the molybdenum-blue method on a continuous flow injection analyzer (CFA-3000) (RealScience Technology, Beijing, China) [22]. For stomatal characterization, a 1 cm × 1 cm segment from the midrib-proximal region of dried leaves underwent epidermal maceration. Samples were immersed in a 1:1 (*v*/*v*) solution of 20% nitric acid and 20% chromic acid for 4–12 h (extended to 30 d for sclerophyllous taxa, e.g., *Rhododendron* spp. and select *Cyclobalanopsis* spp.). Macerated epidermal layers were rinsed, fixed in FAA solution (formalin: acetic acid: 70% ethanol, 5:5:90 by volume), and mounted on glass slides. Stomatal morphology—length (SL), width (SW), perimeter (SC), area (SA), SL/SW ratio, and density (SP; stomata mm^−2^)—was quantified using Motic Images Advanced 3.2 software under 400× magnification.

#### 2.4.3. Derived Trait Calculations

Key leaf trait parameters were computed using the following equations:

Specificleafarea (SLA):

(1) $$SLA=LA/LDM[m^{2}\cdot {\mathrm{kg}}^{-1}]$$

Leafmassperarea (LMA,equivalenttoSLW):

(2) $$LMA=LDM/LA[g\cdot m^{-2}]$$

Leaf dry matter content (LDMC):

(3) $$LDMC=LDM/LFM[g\cdot g^{-1}]$$

Leafmoisturecontent (LMC):

(4) $$LMC=(LFM-LDM)/LFM[g\cdot g^{-1}]$$

### 2.5. Climatic Data Acquisition and Processing

Climatic data were obtained from the WorldClim platform (version 2.1; http://www.worldclim.org). A total of three climatic variables were extracted: mean solar radiation (SR); annual mean temperature (AMT), which is represented as Bio1 in this study; and annual precipitation (AP), represented as Bio2.

These climate factors, combined with supplementary environmental variables, were analyzed to examine their correlations with the functional traits of *T. sinense* populations across their geographical distribution. 

### 2.6. Data Statistics and Analysis

#### 2.6.1. Parameters of Community Ecological Characteristics

(1) Species Importance Assessment

The comprehensive importance value (IV) for each species was calculated as

(5) IV=(RA+RF+RDo)/3

Relative Abundance (RA): The proportion of individuals of a given species relative to the total number of individuals of all species across all quadrats, calculated as

(6) $$RA=(\frac{Number\;of\;individuals\;of\;a\;species}{Total\;individuals\;of\;all\;species}\times 100\%)$$

Relative Frequency (RF): The proportion of quadrats where a given species occurs, calculated as

(7) $$RF=(\frac{Frequency\;of\;a\;species}{Sum\;of\;frequencies\;for\;all\;species})\times 100\%$$

Relative Dominance (RDo): The proportion of basal area (calculated from DBH) of a given species relative to the total basal area of all species, representing its spatial occupancy:

(8) $$RDo=(\frac{Dominance\;of\;a\;species}{Total\;dominance\;of\;all\;species})\times 100\%$$

(2) Niche Breadth Estimation (Levins Index, LI)

(9) $$LI=\frac{1}{\sum_{j=1}^{r}p_{ij}^{2}}$$

where P_ij_ = n_ij_/N_i_, with n_ij_ representing the IV of species i in quadrat j, N_i_ as total occurrences of species i across r quadrats.

(3) Interspecific Niche Overlap (Pianka Index, PI)

(10) $$PI=\sum_{j=1}^{r}P_{ij}P_{kj}\sqrt{\sum_{j=1}^{r}P_{ij}^{2}\sum_{j=1}^{r}P_{kj}^{2}}$$

where the Pianka index is the niche overlap index of the species i and species k, which varies between 0 and 1. The closer the value is to 1, the deeper the overlap is, until 1 indicates that the two species’ ecological niche completely overlap.

#### 2.6.2. Functional Trait Analysis

(1) Variation in Traits Among Populations and Between Species

Initially, we evaluated whether different functional traits meet the assumptions of normality and homogeneity of variance. Sample szies for *T. sinense* and its associated species in each community ranged from 44 to 85. If both assumptions are met, we proceeded with a one-way ANOVA; if not, we opted for non-parametric alternatives. Normality was tested using the Kolmogorov–Smirnov test (as implemented by R’s ks.test function), and variance homogeneity was assessed through Bartlett’s test (using R’s bartlett.test function) [23] and Levene’s test (from the car package’s leveneTest function). Parametric comparisons were conducted using the aov function for one-way ANOVA, whereas non-parametric comparisons were performed with the kruskal.test function.

(2) Trait-Environment Correlations

We useed R’s ggplot2 (version 3.5.1) and ggcorrplot packages (version 0.1.4.1) to create correlation heatmaps that illustrate the relationships between functional trait indicators and environmental factors [24]. Spearman’s rank correlation coefficient was applied due to its robustness to outliers and non-linear monotonic relationships. This analysis aims to identify key environmental drivers that influence the functional traits of *T. sinense*.

(3) Principal Component Analysis (PCA)

Considering the high dimensionality and intercorrelation among functional trait indicators, we conducted dimensionality reduction through PCA. The resulting principal components allowed for a comprehensive evaluation of various *T. sinense* populations [25]. Separate PCA analyses were carried out for functional trait indicators and environmental factors, employing the psych package (version 2.6.5) in R to maintain a distinct examination of biological characteristics and environmental variables.

#### 2.6.3. Phylogenetic Analysis

(1) Phylogenetic Tree Construction

Species documented within the study area were systematically identified, quantified, and taxonomically validated against The Plant List database [26,27]. Taxonomic classifications were organized hierarchically (family/genus/species) to standardize nomenclature. To place species with a robust phylogenetic framework, a phylogenetic tree was subsequently reconstructed using the V.PhyloMaker2 package in R software (version 4.3.1), employing the GBOTB backbone. This approach is suitable for community-level analysis despite limited species-level resolution.

(2) Phylogenetic Signal Quantification

To evaluate the evolutionary conservatism of functional traits, Blomberg’s K statistic was calculated using the phylosignal package (version 1.3.1) in R. This metric quantifies the phylogenetic signal by comparing the observed trait variance to expectations under a Brownian motion model. Since K values alone provide limited resolution, complementary significance tests were performed using the phyloCorrelogram and lipaMoran functions. These methods evaluate Moran’s I-based phylogenetic clustering across different evolutionary depths, thus complementing Blomberg’s K analysis by uncovering patterns of phylogenetic autocorrelation at various taxonomic scales. To enable cross-trait comparison of signal strength, K values were standardized using either Z-score standardization, depending on trait distribution.

(3) Phylogenetic Diversity and Community Structure Analysis

Phylogenetic α-diversity was estimated using Faith’s Phylogenetic Diversity (PD) index, which quantifies the cumulative branch length of species within a community.

To characterize phylogenetic structure, two complementary metrics were calculated: (i) Mean Phylogenetic Distance (MPD), representing the average pairwise evolutionary divergence among all species pairs, and (ii) Mean Nearest Taxon Distance (MNTD), reflecting the mean divergence between each species and its closest relative within the community. These indices were computed via the mpd and mntd functions in the picante package (version 1.8.2). Null model analysis (999 randomizations) standardized MPD values into Standardized Effect Sizes (SESs), where SES > 0 indicates phylogenetic overdispersion (distant relatives) and SES < 0 signifies clustering (close relatives). All workflows utilized rooted phylogenies processed through integrated R packages (version 4.4.2) (tidyverse, devtools, iNEXT.3D) to ensure reproducibility and alignment with community phylogenetics frameworks.

## 3. Results

### 3.1. Community Characteristics

**Figure 2:**
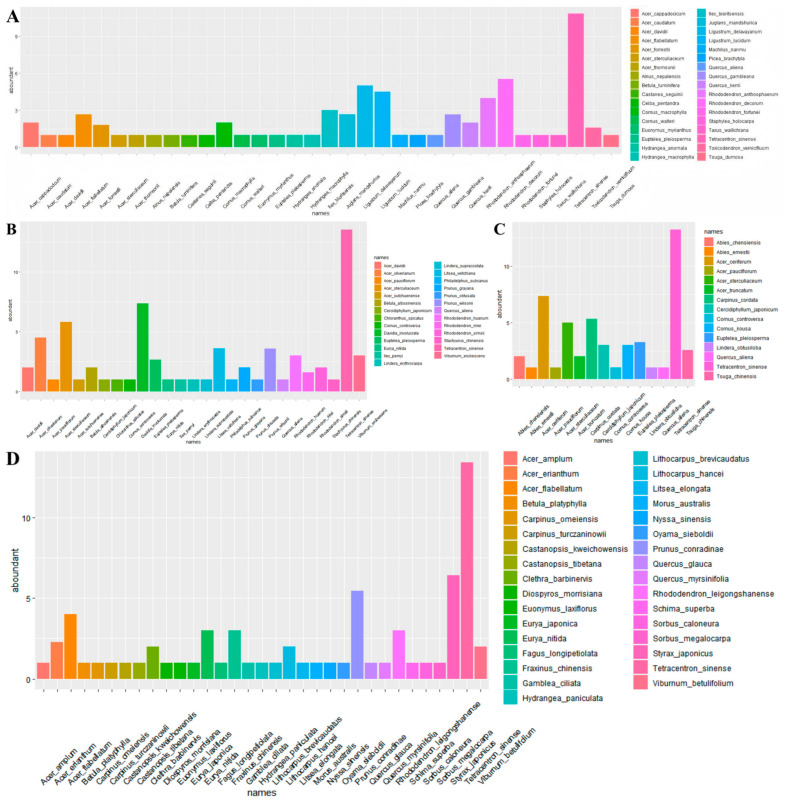
Niche widths in different communities. Note: (**A**) the BMXS community; (**B**) the DFD community; (**C**) the FP community; (**D**) the LGS community.

To characterize the competitive environment and resource utilization strategies of *T. sinense* among populations, we calculated the species importance values (IVs), niche breadth (Levins index), and niche overlap (Pianka index). Across all four studied communities, *Tetracentron sinense* consistently exhibited absolute dominance, with importance values (IVs) ranging from 0.66 to 0.67, indicating its primary control over canopy resources such as light and space. *T. sinense* achieved the maximum Levins Index (LI) values in each community (BMXS = 10.85; DFD = 13.33; FP and LGS also showing the maximum values), confirming its broad niche breadth and extensive capacity to utilize multi-dimensional resources (Figure 2).

Niche overlap analysis using Pianka indices revealed distinct patterns for *T. sinense* across communities. In BMXS, the strongest overlap occurred with *Rhododendron anthosphaerum* (0.72), followed by *Swida walteri* (0.58) and *Cornus macrophylla* (0.52), with all other species showing overlap <0.50. The DFD community exhibited overlap exceeding 0.50 solely with *Davidia involucrata* (0.51). Within FP, the highest overlap was with *Acer fangianum* (0.59), then *Acer ceriferum* (0.57) and *Euptelea pleiosperma* (0.53), while overlap with other species remained below 0.50. In LGS, maximum overlap was observed with *Styrax japonicus* (0.63), followed by *Eurya nitida*, *Fraxinus chinensis*, and *Rhododendron leigongshanense* (all 0.50) (Supplementary Tables S1–S4).

### 3.2. Functional Trait Analysis

- Functional Trait Variation Among Populations

**Figure 3:**
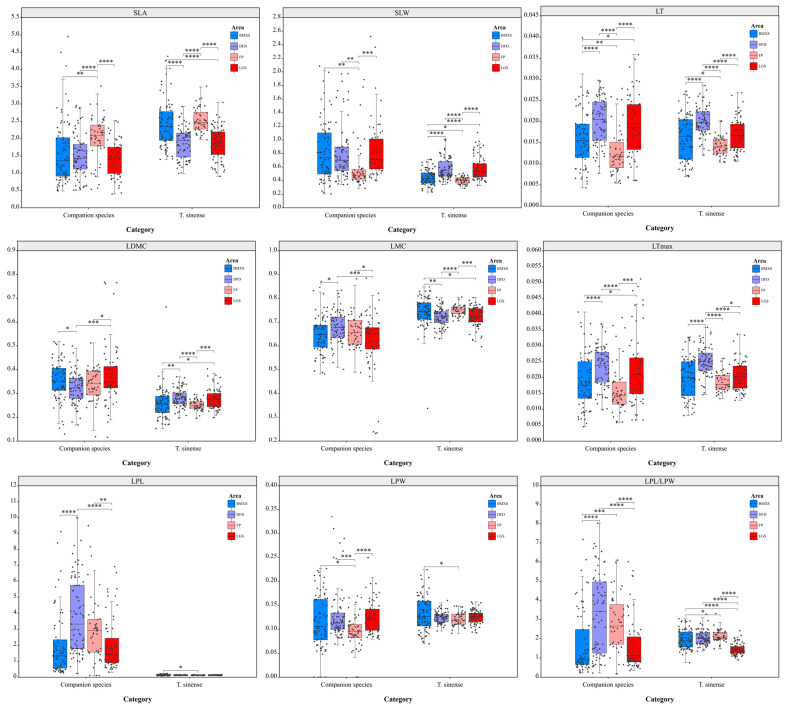

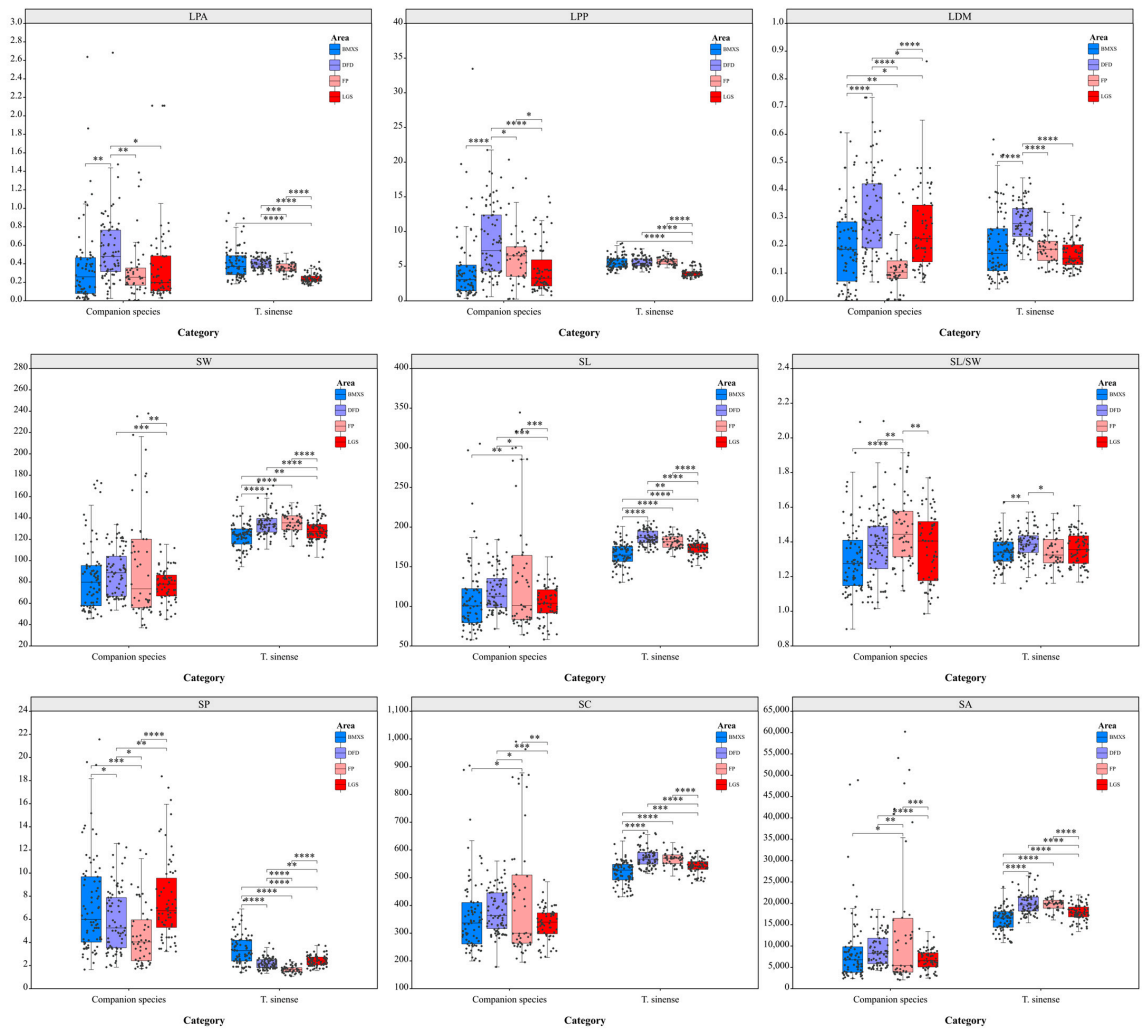

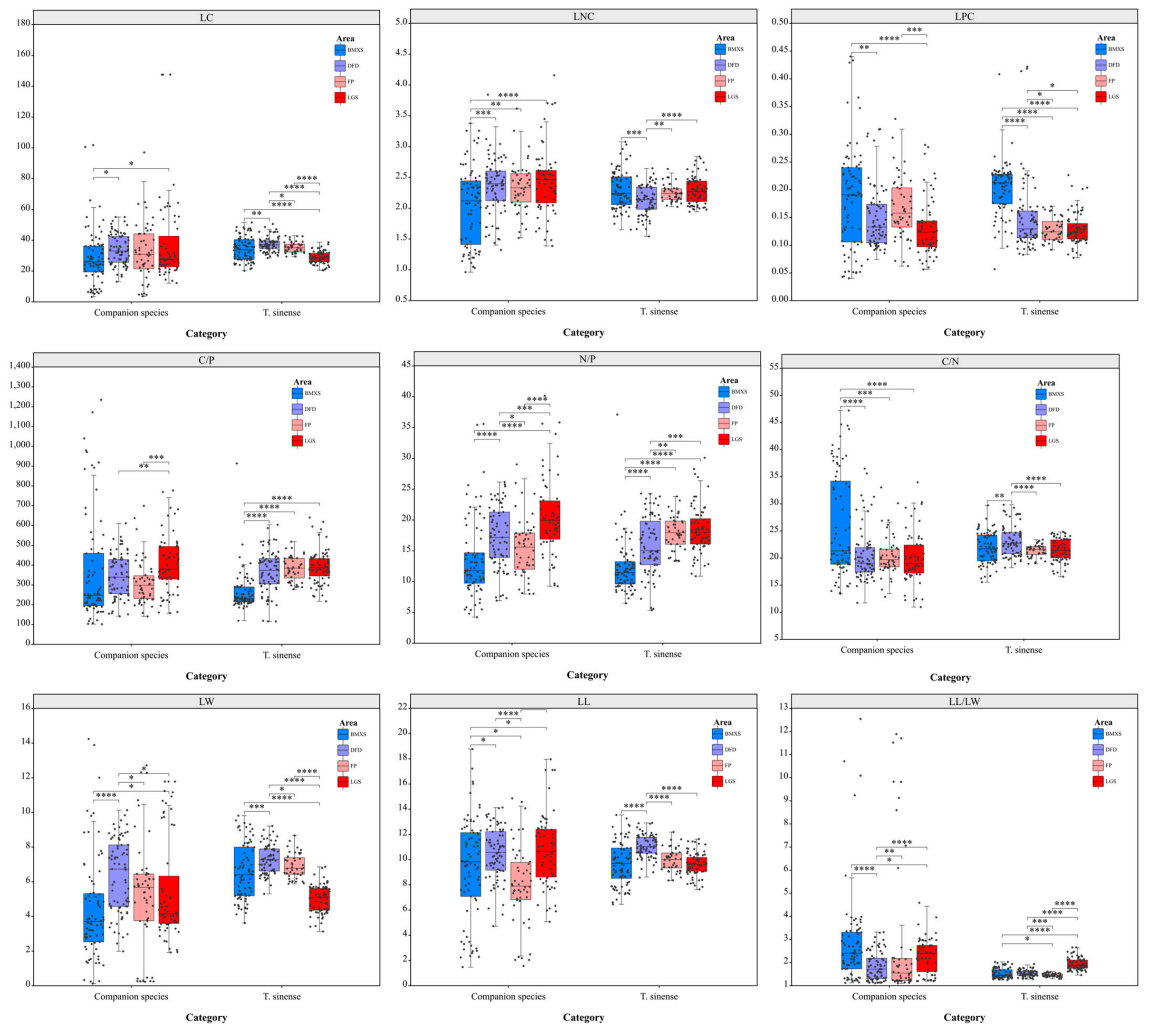

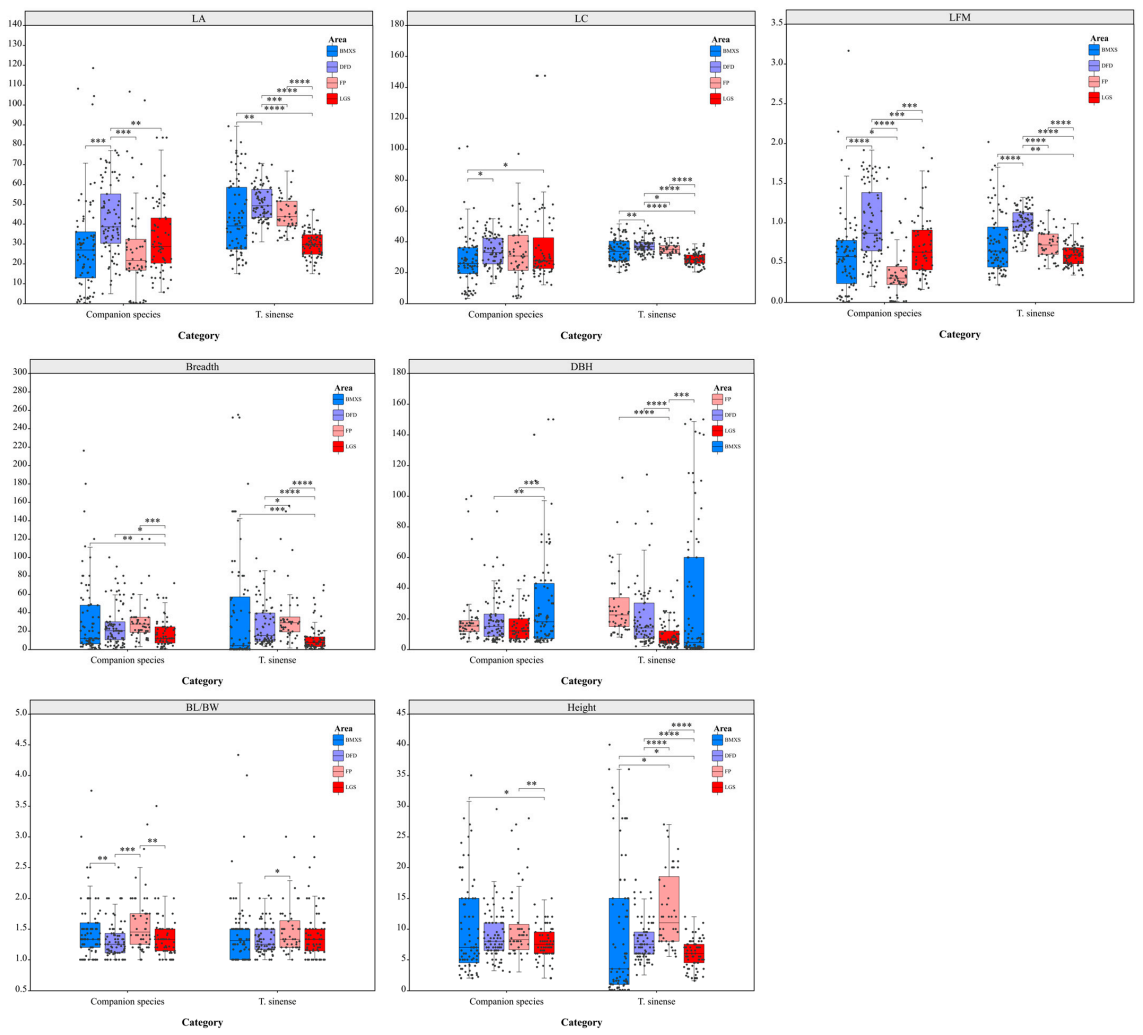
Differences in functional traits between *T. sinense* and its associated species across communities. * *p* < 0.05, ** *p* < 0.01, *** *p* < 0.001, **** *p* < 0.0001 (Tukey’s HSD test). Horizontal lines with asterisks indicate significant differences among sampling sites for the same species category.

The functional traits of *T. sinense* exhibited significant population-level differentiation (Figure 3). The DFD population dominated leaf morphological traits, with the highest leaf area (LA) and leaf length (LL), while the FP population excelled in structural adaptations, including maximum leaf thickness (LTmax), petiole length-to-width ratio (LPL/LPW), canopy width (Breadth), and tree height (Height). Chemical stoichiometry showed community-specific patterns (*p* < 0.05): the LGS population had the highest carbon-to-phosphorus (C/P) and nitrogen-to-phosphorus (N/P) ratios, the BMXS population exhibited optimal leaf phosphorus content (LPC), and the DFD population was characterized by an elevated carbon-to-nitrogen ratio (C/N) and leaf area projection (LPA). Stomatal adaptations further distinguished populations—BMXS had the largest stomatal pore (SP), whereas DFD demonstrated the highest stomatal length/stomatal width (SL/SW).

Overall, the DFD and FP populations exhibited significantly superior trait levels compared to BMXS and LGS, with DFD excelling in leaf expansion (LA) and mechanical support (LDMC), and FP showing advantages in stomatal regulation (SA, SC) and vertical growth (Height). The BMXS population displayed high variability in petiole morphology (LPW, LPP) and stoichiometry (LPC), indicating diverse environmental adaptation strategies, while FP and LGS exhibited greater trait stability.

- Comparison of functional traits between *T. sinense* and its associated species

According to Figure 3, *T. sinense* significantly outperformed its associated species (*p* < 0.05) across four communities in twelve key functional traits: SLA, LWC, LTmax, LPW, SW, SL, SP, SA, LC, C/N ratio, LW, and LP. Exceptions occurred in LT, where mean values in DFD and LGS were lower than those of associated species, and BMXS displayed high variability, suggesting constrained morphological plasticity under competition. Nutrient utilization traits (leaf dry matter content [LDM], SL/SW, N/P, fresh leaf mass [LFM]) were significantly lower in BMXS and LGS compared to associated species. Specifically, N/P < 14 in BMXS indicated nitrogen limitation. For spatial competition traits (LPL/LPW, LPP, LPA, LNC, LPC, DBH, Height), *T. sinense* underperformed relative to associated species in DFD, FP, and LGS, highlighting reduced competitiveness for light and nutrients.

- Trait-Environment Correlations

**Figure 4:**
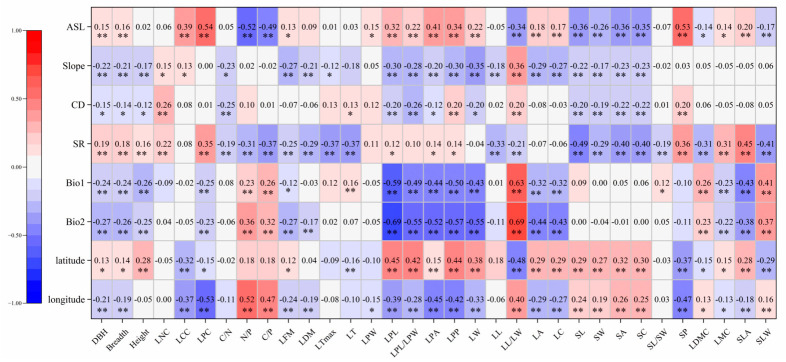
Correlation heat map between functional traits and environmental factors of *T. sinense* at regional scale. Note: Spearman’s rank correlation coefficients were used. * *p* < 0.05, ** *p* < 0.01. Squares closer to deep red indicate stronger positive correlations, while squares closer to deep blue indicate stronger negative correlations. The same applies to the figures below.

According to Figure 4, functional traits of *T. sinense* exhibited distinct correlations with environmental factors. Elevation was negatively correlated with N/P, C/P, LL/LW, stomatal length (SL), SW, SLW, and LDMC, but positively correlated with DBH, Breadth, leaf carbon content (LCC), LPC, leaf width (LW), LA, and SLA. Canopy closure was also negatively correlated with DBH, Breadth, and C/N, and positively correlated with leaf nitrogen content (LNC) and leaf thickness (LT). Steeper slopes were associated with a decrease in plant size, LPA, petiole circumference (LPP), and LA, but an increase in LNC and LL/LW. Higher solar radiation was positively correlated with DBH, Height, leaf moisture content (LMC), and SLA, while negatively correlated with nutrient ratios and LDMC. Temperature-related bioclimatic variables (Bio1: annual mean temperature; Bio2: temperature seasonality) had a negative impact on plant size and leaf area traits, but positively correlated with N/P, C/P, and LT. Higher latitudes was positively correlated with DBH, Height, and LA, but negatively correlated with LCC and LDMC; eastern longitudes was negatively correlated with structural traits while positively correlated with N/P, C/P, and SD (SL/SW).

- PCA-Based Functional Trait Variation in *Tetracentron sinense* Populations

**Figure 5:**
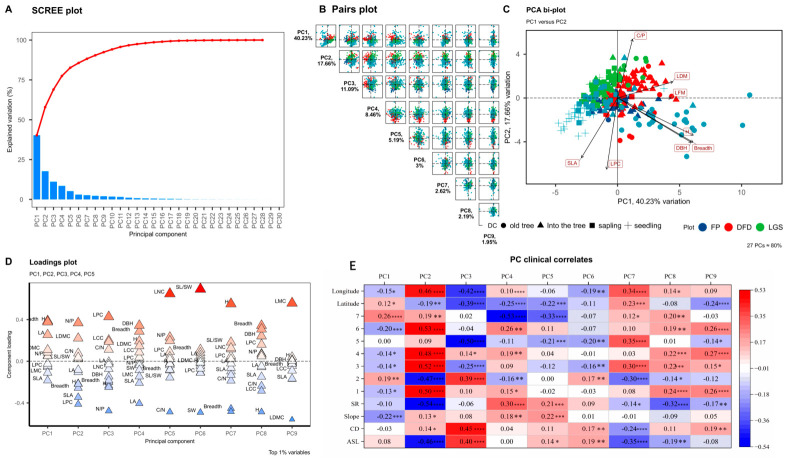
Complex principal component analysis of *T. sinense*. Note: (**A**) Scree plot of principal component analysis of functional traits of *T. sinense*; (**B**) Pair plot of principal component analysis of functional traits of *T. sinense*; (**C**) Biplotof principal component analysis of functional traits of *T. sinense*; (**D**) Loading plot of principal component analysis of functional traits of *T. sinense*; (**E**) Heatmap of correlations between principal components of functional traits and climatic indicators of *T. sinense.* * *p* < 0.05, ** *p* < 0.01, *** *p* < 0.001, **** *p* < 0.0001.

Analysis of 35 functional traits across *T. sinense* populations yielded eight principal components (eigenvalues > 1) explaining 90.44% cumulative variance (Figure 5A,B). PC1-PC4 dominated trait variation (77.44% cumulative contribution), with individual contributions of 40.23% (PC1), 17.66% (PC2), 11.09% (PC3), and 8.46% (PC4). PCs 5–8 accounted for residual variation (5.19–2.19%). Trait loading patterns (Figure 5C,D) revealed distinct associations: PC1 correlated positively with DBH and tree height (indicating larger trees with smaller SLA); PC2 linked to higher N/P ratios and lower phosphorus; PC3 associated with higher phosphorus and lower N/P; and PC4 reflected larger DBH with smaller leaves. Subsequent PCs (5–8) highlighted relationships with leaf nitrogen (PC5), stomatal shape (PC6), tree height versus carbon content (PC7), and crown breadth (PC8).

Environmental correlations revealed hierarchical controls (Figure 5E): PC1-PC3 responded to macroclimatic gradients, with PC1 weakly associated with thermal parameters (Bio1/Bio2), PC2 strongly temperature-dependent (Bio1/Bio2) but light-avoidant (SR), and PC3 elevationally specialized (ASL). Microhabitat factors (Slope, CD) weakly influenced PC4, while solar radiation (SR) drove PC7-8 variation. Population distributions exhibited community-specific adaptation (Figure 5C): FP samples clustered in PC3/PC5/PC7-negative space, suggesting stress tolerance; DFD dominated PC2-positive/PC4-negative axes, indicating high-light adaptation; LGS combined PC1-negative (small size) with PC2/PC4-positive traits (high N/P); and BMXS occupied PC2-negative/PC3-positive space, reflecting phosphorus-efficient ecotypes.

### 3.3. Phylogenetic Analysis of Tetracentron sinense Communities

- Phylogenetic Tree Construction

**Figure 6:**
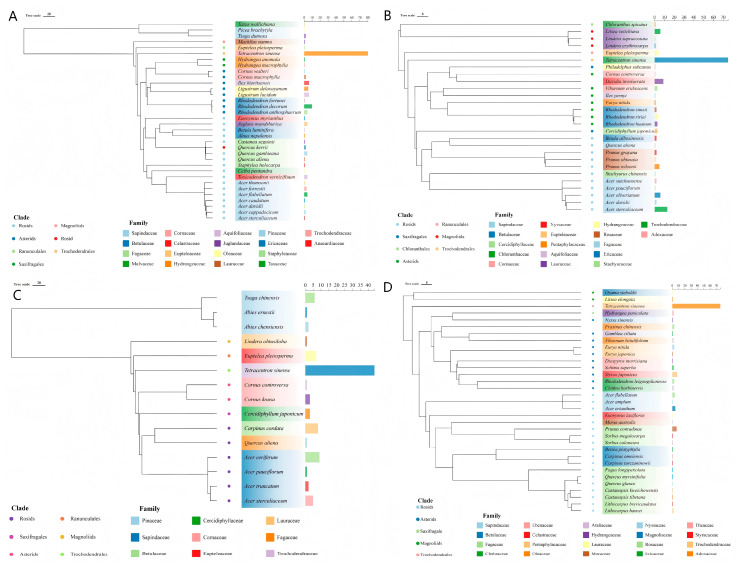
Phylogenetic tree. Note: (**A**) Phylogenetic tree of BMXS community; (**B**) Phylogenetic tree of DFD community; (**C**) BPhylogenetic tree of FP community; (**D**) Phylogenetic tree of LGS community.

As shown in Figure 6A–D, the four communities in this study recorded a total of 542 plant specimens belonging to 32 families, 47 genera, and 97 species. Among the associated species of *T. sinense*, plants from the Moraceae family were most abundant, followed by those from Ericaceae, Fagaceae, Rosaceae, Betulaceae, Oleaceae, Pinaceae, and Lauraceae families. Additionally, plants with closer phylogenetic relationships to *T. sinense* include *Euonymus myrianthus*, while those with more distant phylogenetic connections are predominantly from the Anispermaceae, Hydrangeaceae, and Pinaceae families. The phylogenetic tree analysis reveals that the branch of *T. sinense* extends significantly longer than those of other plants, and it branches off distinct lineages from Pinaceae, Styracaceae, and Hydrangeaceae.

- Phylogenetic signals across community scales

**Figure 7:**
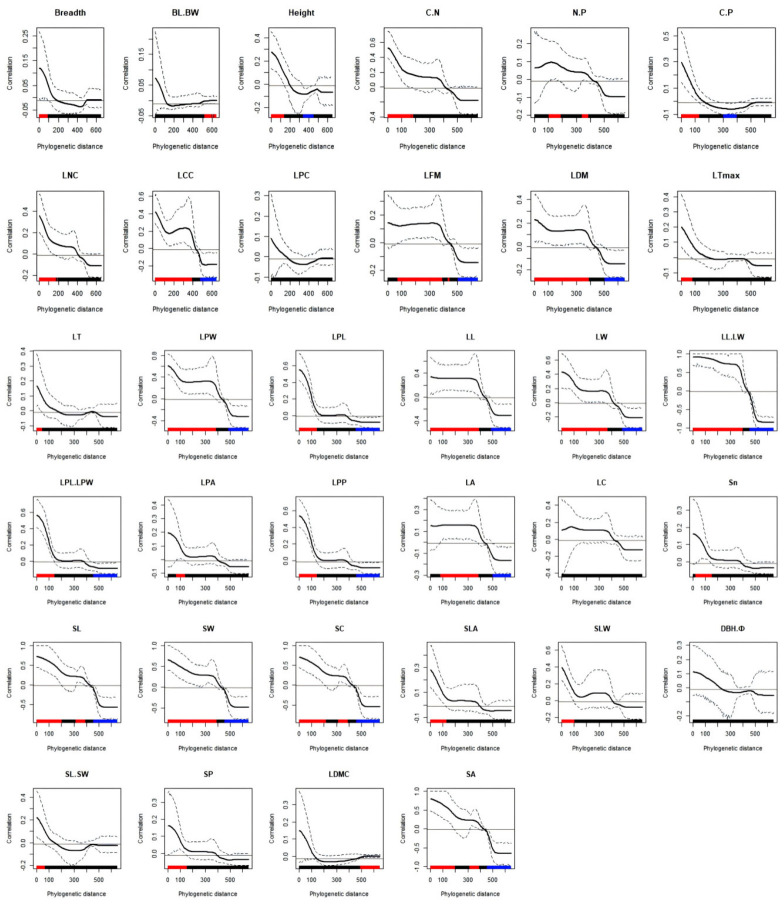
Phylogenetic correlogram of functional traits at large regional scales. Note: The solid black line represents Moran’s I values; the dashed black lines indicate the lower and upper bounds of the 95% confidence envelope. The horizontal black line denotes the expected value of Moran’s I under the null hypothesis of no phylogenetic autocorrelation. The color bar indicates whether autocorrelation is significant based on the confidence interval: red represents significant positive autocorrelation, black represents non-significance, and blue represents significant negative autocorrelation.

Figure 7 shows the trend of Moran’s I index with increasing phylogenetic distance. Breadth, C/N, LNC, LTmax, LT, LA, Sn, SLA, SLW, SL/SW, and SP exhibit significant positive autocorrelation only at short phylogenetic distances. BL/BW and LDMC show positive autocorrelation only at large phylogenetic distances. LPC, LC, and DBH do not show any significant correlation. Height and C/P exhibit positive autocorrelation at short phylogenetic distances, transition to negative autocorrelation at intermediate distances, and then show no significant correlation with further increasing phylogenetic distance. LCC, LFM, LDM, LPW, LPL, LL, LW, LL/LW, LPL/LPW, LPP, LA, SL, SW, SC, and SA show significant positive autocorrelation at short phylogenetic distances, no correlation at intermediate distances, and significant negative correlation at large distances.

As shown in Supplementary Figure S1, plants in the Pinaceae, Myrtaceae, and Ericaceae families exhibit strong phylogenetic signals across most functional traits. The Pinaceae family demonstrates robust phylogenetic signals for Breadth, DBH, H, LNC, C/N, C/P, SP, LDMC, SLA, SLW, and LWC. Myrtaceae plants show significant phylogenetic signals in SLW, SP, LC, LA, LW, and petiole characteristics. Ericaceae plants exhibit pronounced phylogenetic signals regarding leaf nutrient content, weight, thickness, and SLA. Regionally, the phylogenetic signals of water fir traits are less prominent compared to other plant groups. Among the 35 selected functional traits, LA, LW, SLA, and SLW more readily demonstrate significant phylogenetic signals.

- Phylogenetic Signal Distribution in *T. sinense* Communities

BMSM community (Supplementary Figure S2A): Plants from the families Pinaceae, Taxaceae, Sapindaceae, Cornusaceae, and Ericaceae exhibited significant phylogenetic signals across multiple functional traits. Pinaceae and Taxaceae (gymnosperms) showed strong phylogenetic signals in shared traits. Sapindaceae displayed particularly robust phylogenetic signals in leaf structure, petiole morphology, and stomatal function. *T. sinense* demonstrated weaker phylogenetic signals compared to other species, with significant signals observed in LWC, SLA, SP, and LL/LW.

DFD community (Supplementary Figure S2B): Pinaceae plants showed strong phylogenetic signals in petiole morphology (LW, LP/LW), stomatal traits (SW, SP), and leaf element content (LC). Ericaceae exhibited strong signals in LNC, LCC, C/N, N/P, LT, LPW, LPL/LPW, LL, LW, SL/SW, SLA, and SLW. *T. sinense* presented generally weak phylogenetic signals overall, with only LWC showing relatively significant conservation.

FP community (Supplementary Figure S2C): Pinaceae plants demonstrated strong phylogenetic signals in SLA, SLW, SL, SW, SC, SA, LNC, LCC, C/N, N/P, and traits in petiole, leaf blade, and leaf mass. Fagaceae conserved petiole type, leaf type, and stomatal type traits. Cornusaceae showed significant signals in leaf element content. *T. sinense* lacked strong phylogenetic signals in most traits but displayed moderate conservation in N/P ratio, LPW, LL, LW, LL/LW, SLA, and SLW.

LGS community (Supplementary Figure S2D): Fagaceae plants exhibited strong phylogenetic signals in LPL, LPW, LPA, LPP, LW, LL/LW, LA, LC, SL, SW, and SP. Another Fagaceae group conserved LDMC, SLW, SLA, LWC, SL/SW, LL/LW, LPW, and LDM. Pentaphylacaceae showed significant signals in LNC, LPC, C/N, C/P, LFM, LL, LA, LC, SLA, and SLW. *T. sinense* displayed no dominant phylogenetic signals but showed localized conservation in Breadth, C/P, LDM, LL, LL/LW, SL/SW, LDMC, SLW, and LWC.

- Phylogenetic Structure in *T. sinense* Communities

According to Table 1, at the community level, species richness was highest in the BMXS community and lowest in the FP community. The observed modularity (mod.obs) was greatest in the FP community, followed by the BMXS community, with the LGS community exhibiting the smallest modularity. While mean phylogenetic distance (MPD) values were comparable across the communities, their standard deviations differed significantly: the FP community showed the highest standard deviation (44.2), and the BMXS community the lowest (24.8). Standardized effect size (SES) values were negative for the DFD and LGS communities but positive for the BMXS and FP communities. Critically, the SES for the FP community exceeded 1.96, indicating significant phylogenetic overdispersion, while the LGS community had an SES below −1.96, signifying strong phylogenetic clustering.

## 4. Discussion

- Ecological Dominance and Interspecific competition pressures of *T. sinense*

The importance value (IV) reflects a species’ ecological significance, while niche breadth indicates its environmental adaptability, spatial distribution, and competitive potential. A broader niche breadth generally implies stronger resource exploitation capacity and higher environmental resilience [28]. Across all four surveyed communities, *Tetracentron sinense* consistently dominated the arbor layer, exhibiting the highest IV and niche breadth, confirming its role as a constructive species that regulates light interception and nutrient utilization to shape community structure.

Niche overlap (Pinka index) quantifies interspecific resource competition [29]. In each community, 1–3 dominant tree species (e.g., *Rhododendron anthosphaerum* in BMXS and *Styrax japonicus* in LGS) showed substantial niche overlap with *T. sinense* (PI ≥0.5), sharing similar resource requirements for light, soil nutrients and space, leading to intense interspecific competition. Among them, the BMXS community showed the highest niche overlap (0.72), indicating severe resource competition and potential constraints on the growth of *T. sinense*.

Interspecific competition directly affects the population dynamics of *T. sinense*. Sympatric species with similar life forms and resource requirements (e.g., *Rhododendron anthosphaerum* and *Styrax japonicus*) can outcompete *T. sinense* for light, soil nutrients, and growing space, thereby inhibiting its growth and reproduction. Long-term intense competition may lead to niche contraction of *T. sinense*, reducing its population size and distribution range. This, combined with its narrow ecological amplitude and low reproductive capacity, further increases the risk of population decline, highlighting the need for targeted conservation measures to mitigate competitive pressures.

- Variation in functional traits and adaptation among *T. sinense* populations

Plant functional traits mediate organism–environment interactions and reflect evolutionary resource allocation strategies [30]. For *T. sinense*, these traits are stable phenotypic expressions of genetically based variation filtered by long-term natural selection [31], with population-level differentiation indicating evolved adaptive strategies [32]—coordinated adjustments in morphology, physiology, and stoichiometry.

Leaf trait divergence correlates with light competition and nutrient limitation. The DFD population (1100 mm precipitation; shaded, coexisting with *Davidia involucrata*) has larger leaf area (LA) and specific leaf weight (SLW) for light capture, paired with high leaf dry matter content (LDMC) and photosynthetic rates to balance resource use. In contrast, the LGS population (14.3 °C, 1600 mm precipitation, growing seasons > 230 days) shows elevated C/P and N/P ratios and reduced morphological indices, reflecting phosphorus limitation [33].

Stoichiometric analysis reveals contrasting nutrient constraints: BMXS, characterized by a N/P ratio of less than 14, experiences nitrogen limitation, which is linked to a mean temperature of 8.4 °C and postglacial environmental heterogeneity. In contrast, LGS and FP, with N/P ratios greater than 16, face phosphorus limitation, resulting from phosphorus leaching in warm-humid conditions. High-latitude FP (33°38′ N) exhibits increased height and crown width to optimize light capture, while LGS utilizes low C/N litter to accelerate nutrient cycling [34]. Both strategies are growth-oriented.

Stomatal traits, which regulate CO_2_-H_2_O exchange, reflect environment-driven plasticity [35,36]. The inverse relationship between stomatal density (SD) and size indicates that FP’s low SD (associated with 11.5 °C and light competition) reduces transpiration, whereas BMXS’s high SD facilitates gas exchange in cold-arid habitats (<1200 mm precipitation). Solar radiation and canopy closure modulate these traits: high radiation correlates with increased stem thickening (diameter at breast height, DBH) but reduced leaf dry mass per unit area (LDMC); conversely, shade induces leaf thickening (leaf thickness, LT) and elevated leaf nitrogen content (LNC).

Elevational/latitudinal gradients shape adaptive trade-offs: low-altitude populations prioritize light capture (larger LA/SLA), high-altitude populations minimize N/P and C/P ratios for nutrient conservation. High-latitude FP expands leaf area/height to counteract short growing seasons; low-latitude LGS uses elevated C/P ratio for hot-humid stress. DFD/FP’s superior SLA/Height likely stems from glacial refugia roles [18] (preserving genetic diversity), while BMXS’s high phenotypic variability (petiole traits: LPW, LPP, LPC) reflects postglacial adaptability.

Collectively, *T. sinense* trait differentiation arises from environmental pressures and adaptive trade-offs, shaped by historical (glaciation, postglacial recolonization [37]) and modern gradients [38]. The species achieves cross-geographic adaptation via integrated morphostructural, physiometabolic and stoichiometric coordination—insightful for ancient relict persistence.

- Differences in Leaf Functional Traits Between *T. sinense* and its Associated Species

Plants in similar habitats oftern show interspecific variations in leaf functional traits [31]. In the DFD and LGS communities, *T. sinense* had consistently lower average leaf thickness (LT) than its associated species. LT is linked to resource acquisition, water retention [39], and photosynthesis: thicker leaves have higher nitrogen mass per unit weight (Nmass), which enhances light use and water retention [40,41], while thinner leaves favor low-light photosynthesis [42]. Given low effective solar radiation (SR) in DFD and LGS, *T. sinense* may trade off partial nutrient/water retention to optimize dim-light photosynthesis, which needs further validation.

Leaf C, N, and P contents inform terrestrial ecosystem dynamics and global change responses [43]. The N/P ratio reflects nutrient utilization and limitation [44]. In BMXS and LGS, *T. sinense* had lower N/P ratios than associates, indicating inferior nutrient use efficiency. LNC (positively correlated with photosynthetic rate [45]) and LPC (enhancing stress resistance [46]) are key functional indicators. In the DFD, FP, and LGS, *T. sinense* had lower average LNC, LPC, DBH, and height than associates, indicating compromised stress tolerance, photosynthesis, and growth.

In DFD and FP, *T. sinense* also had lower petiole-related traits (length-to-width ratio, perimeter, area) than associates. Petioles support leaves [47], with length affecting light capture [48] and correlating with leaf size. Notably, *T. sinense* had larger average leaf area here, suggesting an adaptive strategy of shorter petioles supporting larger leaves for light competition.

- Comprehensive Evaluation of *T. sinense* Populations Across Regions

Principal component (PC) analyses of functional traits in *T. sinense* populations reveal multidimensional adaptive strategies shaped by resource trade-offs and environmental filtering. PC1 (40.23% variance), positively loaded with DBH and tree height and negatively correlated with SLA in larger trees, reflects whole-plant structural investment consistent with carbon-conservative strategies under high light [49,50]. PC3 (11.09% variance) shows an inverse relationship between N/P ratio and LPC, indicating adaptive adjustments in phosphorus acquisition under nutrient limitation [51]. PC2 (17.66% variance) denotes stress avoidance via elevated N/P ratios and reduced SLA, suggesting phosphorus limitation drives nitrogen-enriched, structurally reinforced leaves. The hierarchical PC organization (macroscale growth traits PC1–PC4; microscale leaf adjustments PC5–PC8) supports the whole-plant economics spectrum theory.

Population distribution along PC axes reflects habitat-driven specialization. The FP population (negative PC3, PC5, PC7) exhibits stress-tolerant traits (constrained vertical growth, low leaf nitrogen, limited crown development) for high-altitude resource conservation. BMXS populations (negative PC2, positive PC3) display phosphorus-efficient traits (high LPC, low N/P) and high SLA, optimizing photosynthesis under high radiation. DFD populations (positive PC2, negative PC4) have high N/P ratios and reduced leaf area, adapting to shaded understory light competition via nitrogen-enhanced photosynthesis. These validate the trait-environment framework, with SLA and N/P as key environmental filtering indicators.

The trade-off between PC2 (slow growth, stress tolerance) and PC3 (fast growth, nutrient acquisition) is a central adaptive challenge. Populations like LGS (negative PC1, positive PC2) balance reduced structural growth with nitrogen-enriched leaves to cope with phosphorus limitation [52]. This multi-trait covariation highlights *T. sinense*’s evolutionary plasticity, enabling persistence across elevational and microclimatic gradients via context-dependent resource prioritization.

- Shaping of Functional Traits by Evolutionary History

Functional trait evolution is jointly driven by environmental filtering and phylogenetic legacy, with structural and functional traits generally evolving coordinately under environmental pressures [53]. Integrating both selective regimes and phylogenetic context is therefore critical for disentangling plant adaptive evolution [54,55]. Our results reveal significant phylogenetic signals in most functional traits of *T. sinense* communities, including leaf morphological (LA, LW, SLA) and physiological (LNC, C/N) traits, demonstrating that interspecific trait variation is primarily constrained by ancestral evolutionary history [56].

Phylogenetic signal strength is taxon-specific: Pinaceae, Sapindaceae and Ericaceae display stronger signals, conferring high trait conservatism within closely related species, whereas basal BL/BW and LDMC only exhibit phylogenetic dependence in terminal clades. This taxonomic differentiation implies that regional trait covariation patterns are phylogenetically regulated.

Phylogenetic signal distribution further differs across community types. Strong signals were detected in Pinaceae, Taxaceae, Sapindaceae, Cornaceae and Ericaceae in BMXS communities; solely in Ericaceae in DFD communities; in Pinaceae, Sapindaceae, and Cornaceae in FP communities; and in Sapindaceae, Fagaceae, and Pentaphylacaceae in LGS communities, indicating pronounced phylogenetic constraints on trait evolution in these taxa. By contrast, most traits of *T. sinense* show weak phylogenetic signals despite partial evolutionary conservatism. At the regional scale, LA, LW, SLA, and SLW are mainly shaped by phylogenetic legacy, whereas local-scale signals (predominantly in LGS and FP communities) are concentrated in LWC, N/P, leaf shape ratios (LL/LW, SL/SW) and dry-matter traits (LDM, LDMC). Collectively, leaf economic trait modules represent the core unit of evolutionary conservation for *T. sinense*, and community-dependent shifts in phylogenetic signals reflect interactive effects between environmental selection and evolutionary history on trait divergence.

- The impact of evolutionary history on the adaptability of *T. sinense* populations

Standardized Effect Size (SES)-based phylogenetic analyses revealed divergent assembly patterns across *T. sinense* communities. FP communities showed significant overdispersion (SES = 1.9733 > 1.96), with a marginal trend in BMXS communities (SES = 0.6023), mechanistically driven by niche similarity-induced competitive exclusion favoring distantly related taxa coexistence [39]. In contrast, LGS communities exhibited significant clustering (SES = −2.0312, SES < −1.96), and DFD communities a clustered tendency (SES = −1.3951), reflecting habitat filtering that assembles closely adapted, phylogenetically related species [57]. Similar habitat filtering processes, driven by temperature and precipitation, have been shown to shape the diversification of East Asian wild grapes [58].

Notably, *T. sinense* community assembly is shaped by contrasting dominant processes: competitive exclusion mediates niche differentiation in BMXS and FP communities, while evolutionary legacy-associated habitat filtering drives co-occurring species’ adaptive convergence in LGS and DFD communities.

## 5. Conclusions

This study demonstrates that *Tetracentron sinense* effectively overcomes phylogenetic niche conservatism through remarkable trait plasticity, enabling divergent adaptive strategies across latitudinal gradients. Despite strong phylogenetic constraints on leaf economic traits and niche conservatism within glacial refugia, populations reconfigure their trait networks to mitigate environmental limitations. The species maintains competitive dominance despite significant niche overlap with sympatric taxa, intensifying resource competition and driving niche differentiation in some communities. Populations employ context-dependent strategies involving metabolic adjustments, structural trade-offs, and evolutionary resilience, as supported by multivariate trait–environment correlations and phylogenetic community structure analyses. However, two key limitations exist: lack of genetic diversity data and investigation of only four populations. We thus propose protecting multiple *T. sinense* populations to conserve trait diversity, critical for sustaining the species’ adaptive potential amid ongoing environmental changes. This study highlights plasticity’s role in overcoming phylogenetic limitations and promoting adaptation to diverse environments, offering important insights for protecting this endangered relict species against continuous environmental changes.

## Figures and Tables

**Table 1:** MPD indices at regional scale.

MDP	BMXS	DFD	FP	LGS
ntaxa	34	37	15	33
mod.obs	296.5773	239.4318	368.9348	229.453
mpd.rand.mean	281.6129	282.4128	281.6325	281.6582
mpd.rand.sd	24.8442	30.8096	44.2425	25.7012
mpd.obs.rank	637	124	958	31
mpd.obs.z	0.6023	−1.3951	1.9733	−2.0312
mpd.obs.p	0.673	0.124	0.958	0.031

Note: In this table, “ntaxa” refers to the number of species in each community; “mod.obs” represents the observed value of modularity; “mpd.rand.mean” denotes the mean MPD value derived from the null model; “mpd.rand.sd” indicates the standard deviation of MPD values from the null model; “mpd.obs.rank” reflects the rank of the observed value within the null model distribution; “mpd.obs.z” corresponds to the standardized effect size (SES) value; “mpd.obs.p” represents the quantile (i.e., *p*-value) of the observed value in the null model results.

## Data Availability

The raw data supporting the conclusions of this article will be made available by the authors on request.

## Supplementary Materials

The following supporting information can be downloaded at https://www.mdpi.com/article/10.3390/biology15120915/s1, Figure S1: Standardized phylogenetic signals of functional traits across regions; Figure S2: Standardized phylogenetic signals of functional traits; Table S1: Niche overlapping Pianka index in BMXS community; Table S2: Niche overlapping Pianka index in DFD community; Table S3: Niche overlapping Pianka index in FP community; Table S4: Niche overlapping Pianka index in LGS community.
