# Supplementary material for: Latitudinal Adaptive Strategies of Tetracentron sinense: Insights from Functional Traits and Phylogenetic Conservatism
Source: Biology (Basel). 2026 Jun 11;15(12):915. doi: 10.3390/biology15120915 (PMC13295673; doi:10.3390/biology15120915)
Supplement: Supplementary file 1 [file biology-15-00915-s001.zip › biology-4302912-supplementary.pdf]

Supplementary Figure

Supplementary Figure S1 Standardized phylogenetic signals of functional traits across regions.

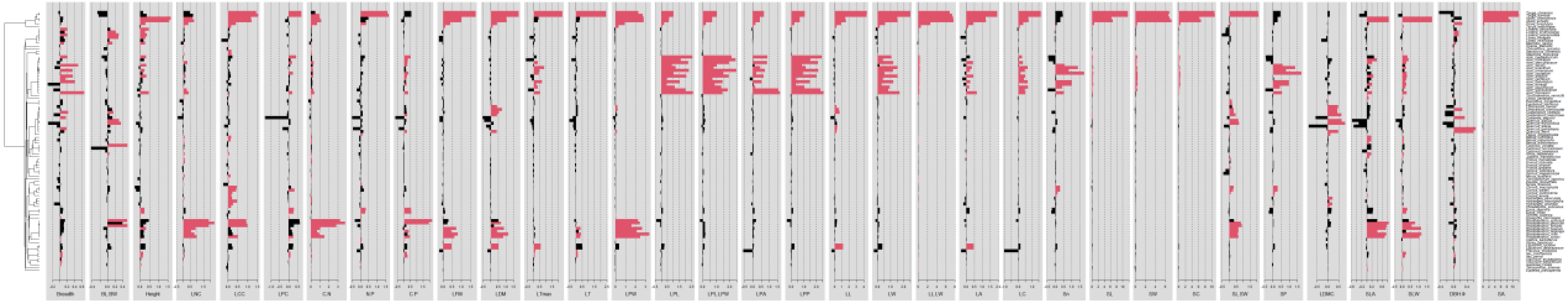

Note: The left panel shows the phylogenetic tree of 96 species, with bars indicating p<0.05 highlighted in red in the histogram, and the right panel displays the scientific names of the plants. The same conventions apply hereafter.

Supplementary Figure S2 Standardized phylogenetic signals of functional traits.

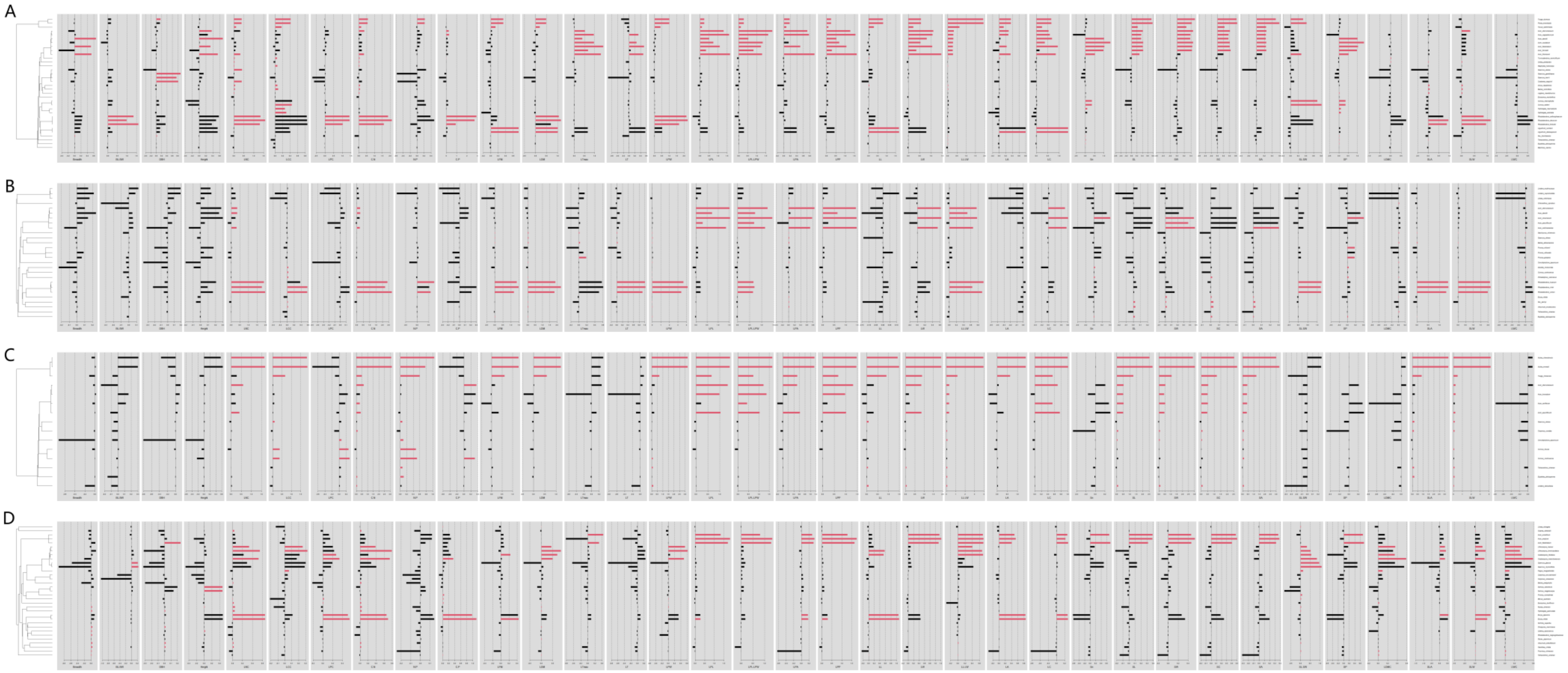

Note: A: BMXS community; B: DFD community; C: FP community; D: LGS community

Supplementary Table

Table S1 Niche overlapping Pianka index in BMXS community

	1	2	3	4	5	6	7	8	9	10	11	12	13	14	15	16	17	18	19	20	21	22	23	24	25	26	27	28	29	30	31	32	33
2	0.71																																
3	0.00	0.00																															
4	0.00	0.00	0.00																														
5	0.00	0.00	0.00	0.00																													
6	0.00	0.00	0.00	0.00	0.00																												
7	0.71	0.00	0.00	0.00	0.00	0.00																											
8	0.00	0.00	0.00	0.00	0.00	0.00	0.00																										
9	0.00	0.00	0.00	0.00	0.45	0.00	0.00	0.00																									
10	0.00	0.00	0.00	0.00	0.00	0.00	0.00	1.00	0.00																								
11	0.00	0.00	0.00	0.00	0.89	0.00	0.00	0.00	0.00	0.00																							
12	0.00	0.00	0.00	0.58	0.00	0.00	0.00	0.00	0.00	0.00	0.00																						
13	0.00	0.00	0.00	0.00	0.00	0.00	0.00	0.00	0.00	0.00	0.00	0.71																					
14	0.00	0.00	0.00	0.00	0.00	0.00	0.00	0.00	0.00	0.00	0.00	0.00	0.00																				

15	0.00	0.00	0.00	0.00	0.89	0.00	0.00	0.00	0.00	0.00	1.00	0.00	0.00	0.00														
16	0.00	0.00	0.00	0.82	0.00	0.00	0.00	0.00	0.00	0.00	0.00	0.71	0.00	0.00	0.00													
17	0.71	0.00	0.00	0.00	0.00	0.00	1.00	0.00	0.00	0.00	0.00	0.00	0.00	0.00	0.00	0.00												
18	0.00	0.00	0.00	0.35	0.00	0.00	0.00	0.87	0.00	0.87	0.00	0.20	0.00	0.29	0.00	0.29	0.00											
19	0.29	0.41	0.00	0.00	0.73	0.00	0.00	0.00	0.00	0.00	0.82	0.29	0.41	0.00	0.82	0.00	0.00	0.00										
20	0.63	0.45	0.00	0.00	0.00	0.45	0.45	0.45	0.00	0.45	0.00	0.00	0.00	0.00	0.00	0.00	0.45	0.39	0.18									
21	0.00	0.00	0.00	0.87	0.00	0.00	0.00	0.35	0.00	0.35	0.00	0.50	0.00	0.00	0.00	0.71	0.00	0.61	0.00	0.16								
22	0.71	1.00	0.00	0.00	0.00	0.00	0.00	0.00	0.00	0.00	0.00	0.00	0.00	0.00	0.00	0.00	0.00	0.00	0.41	0.45	0.00							
23	0.00	0.00	0.00	0.00	0.00	0.00	0.00	0.00	0.00	0.00	0.00	0.00	0.00	0.00	0.00	0.00	0.00	0.00	0.00	0.00	0.35	0.00						
24	0.00	0.00	0.00	0.00	0.00	1.00	0.00	0.00	0.00	0.00	0.00	0.00	0.00	0.00	0.00	0.00	0.00	0.00	0.00	0.45	0.00	0.00	0.00					
25	0.00	0.00	0.00	0.00	0.18	0.00	0.00	0.82	0.41	0.82	0.00	0.00	0.00	0.41	0.00	0.00	0.00	0.82	0.00	0.37	0.29	0.00	0.00	0.00				
26	0.00	0.00	0.00	0.00	0.32	0.00	0.00	0.00	0.71	0.00	0.00	0.00	0.00	0.71	0.00	0.00	0.00	0.20	0.00	0.00	0.00	0.00	0.00	0.00	0.58			
27	0.35	0.00	0.00	0.20	0.45	0.00	0.50	0.00	0.00	0.00	0.50	0.35	0.50	0.00	0.50	0.00	0.50	0.14	0.61	0.22	0.18	0.00	0.00	0.00	0.00	0.00		
28	0.33	0.47	0.00	0.10	0.42	0.47	0.00	0.24	0.00	0.24	0.47	0.00	0.00	0.00	0.47	0.00	0.00	0.20	0.58	0.53	0.17	0.47	0.00	0.47	0.19	0.00	0.24	
29	0.00	0.00	0.00	0.41	0.00	0.00	0.00	0.00	0.00	0.00	0.00	0.00	0.00	0.00	0.00	0.00	0.00	0.29	0.00	0.00	0.35	0.00	0.00	0.00	0.00	0.00	0.50	0.00

30	0.71	0.00	0.00	0.00	0.00	0.00	1.00	0.00	0.00	0.00	0.00	0.00	0.00	0.00	0.00	0.00	1.00	0.00	0.00	0.45	0.00	0.00	0.00	0.00	0.00	0.00	0.50	0.00	0.00				
31	0.00	0.00	0.00	0.41	0.00	0.00	0.00	0.00	0.00	0.00	0.00	0.00	0.00	0.00	0.00	0.00	0.00	0.00	0.00	0.00	0.35	0.00	0.00	0.00	0.00	0.00	0.00	0.24	0.00	0.00			
32	0.29	0.08	0.21	0.27	0.29	0.04	0.33	0.16	0.16	0.16	0.25	0.52	0.58	0.21	0.25	0.16	0.33	0.33	0.47	0.35	0.36	0.08	0.21	0.04	0.29	0.26	0.72	0.42	0.29	0.33	0.04		
33	0.00	0.00	0.95	0.00	0.00	0.00	0.00	0.00	0.00	0.00	0.00	0.00	0.00	0.00	0.00	0.00	0.00	0.00	0.00	0.14	0.00	0.00	0.00	0.00	0.00	0.00	0.00	0.00	0.00	0.00	0.00	0.25	
34	0.71	1.00	0.00	0.00	0.00	0.00	0.00	0.00	0.00	0.00	0.00	0.00	0.00	0.00	0.00	0.00	0.00	0.00	0.41	0.45	0.00	1.00	0.00	0.00	0.00	0.00	0.00	0.47	0.00	0.00	0.00	0.08	0.00

Note: 1. *Acer cappadocicum* var. *sinicum*; 2. *Acer caudatum*; 3. *Acer davidii*; 4. *Acer flabellatum*; 5. *Acer forrestii*; 6. *Acer franchetii*; 7. *Acer thomsonii*; 8. *Alnus nepalensis*; 9. *Betula luminifera*; 10. *Castanea seguinii*; 11. *Ceiba pentandra*; 12. *Cornus macrophylla*; 13. *Swida walteri*;14. *Euonymus myrianthus*;15. *Euptelea pleiosperma*;16. *Hydrangea anomala*;17. *Hydrangea macrophylla*;18. *Ilex bioritsensis*;19. *Juglans mandshurica*; 20. *Ligustrum delavayanum*;21. *Ligustrum lucidum*;22. *Machilus nanmu*; 23. *Picea brachytyla* var. *complanata*; 24. *Quercus aliena*;25. *Cyclobalanopsis gambleana*;26. *Cyclobalanopsis kerrii*;27. *Rhododendron anthosphaerum*; 28. *Rhododendron decorum*; 29. *Rhododendron fortunei*; 30. *Staphylea holocarpa*; 31. *Taxus chinensis*; 32. *Tetracentron sinense*; 33. *Toxicodendron vernicifluum*; 34. *Tsuga dumosa*.

Table S2 Niche overlapping Pianka index in DFD community

	1	2	3	4	5	6	7	8	9	10	11	12	13	14	15	16	17	18	19	20	21	22	23	24	25	26
2	0.00																									
3	0.00	0.00																								
4	0.00	0.26	0.00																							
5	0.00	0.00	0.00	0.00																						
6	0.00	0.00	0.71	0.00	0.71																					
7	0.00	0.35	0.00	0.00	0.00	0.00																				
8	0.00	0.00	0.00	0.00	1.00	0.71	0.00																			
9	0.00	0.35	0.00	0.00	0.00	0.00	1.00	0.00																		
10	0.00	0.64	0.00	0.67	0.00	0.00	0.00	0.00	0.00																	
11	0.00	0.00	0.82	0.00	0.41	0.87	0.00	0.41	0.00	0.00																
12	0.71	0.00	0.00	0.00	0.00	0.00	0.00	0.00	0.00	0.00	0.00															
13	0.00	0.00	1.00	0.00	0.00	0.71	0.00	0.00	0.00	0.00	0.82	0.00														
14	0.71	0.00	0.00	0.00	0.00	0.00	0.00	0.00	0.00	0.00	0.00	0.00	0.00													
15	0.00	0.00	0.00	0.37	0.00	0.00	0.00	0.00	0.00	0.30	0.00	0.00	0.00	0.00												
16	0.45	0.00	0.63	0.00	0.32	0.67	0.00	0.32	0.00	0.10	0.65	0.00	0.63	0.63	0.00											
17	0.00	0.35	0.00	0.00	0.00	0.00	0.00	0.00	0.00	0.30	0.00	0.00	0.00	0.00	0.00	0.00										
18	0.00	0.25	0.00	0.26	0.00	0.00	0.00	0.00	0.00	0.21	0.00	0.00	0.00	0.00	0.00	0.00	0.71									
19	0.00	0.00	0.00	0.37	0.00	0.00	0.00	0.00	0.00	0.30	0.00	0.00	0.00	0.00	1.00	0.00	0.00	0.00								
20	0.00	0.80	0.00	0.42	0.00	0.00	0.00	0.00	0.00	0.68	0.00	0.00	0.00	0.00	0.00	0.00	0.38	0.27	0.00							
21	0.00	0.35	0.00	0.19	0.00	0.00	0.00	0.00	0.00	0.30	0.00	0.00	0.00	0.00	0.00	0.00	0.00	0.00	0.00	0.00						
22	0.00	0.41	0.00	0.64	0.00	0.00	0.00	0.00	0.00	0.35	0.00	0.00	0.00	0.00	0.00	0.00	0.00	0.41	0.00	0.44	0.00					
23	0.00	0.00	0.00	0.70	0.00	0.00	0.00	0.00	0.00	0.38	0.00	0.00	0.00	0.00	0.00	0.00	0.00	0.00	0.00	0.36	0.00	0.18				
24	0.50	0.00	0.00	0.26	0.00	0.00	0.00	0.00	0.00	0.00	0.00	0.71	0.00	0.00	0.00	0.00	0.00	0.50	0.00	0.00	0.00	0.41	0.00			
25	0.00	0.00	0.00	0.00	0.00	0.00	0.00	0.00	0.00	0.00	0.41	0.00	0.00	0.00	0.00	0.00	0.00	0.00	0.00	0.00	0.00	0.00	0.00	0.00		

26	0.38	0.49	0.15	0.47	0.29	0.31	0.34	0.29	0.34	0.56	0.42	0.34	0.15	0.20	0.20	0.40	0.25	0.31	0.20	0.37	0.25	0.34	0.25	0.38	0.44	
27	0.00	0.00	0.00	0.64	0.58	0.41	0.00	0.58	0.00	0.35	0.24	0.00	0.00	0.00	0.00	0.18	0.00	0.00	0.00	0.222	0.00	0.33	0.73	0.00	0.00	0.40

Note: 1. *Acer davidii,* 2. *Acer oliverianum,* 3. *Acer laxiflorum*, 4. *Acer franchetii*, 5. *Acer sutchuenense*, 6. *Betula albosinensis*, 7. *Cercidiphyllum japonicum*, 8. *Chloranthus spicatus*, 9. *Cornus controversa*, 10. *Davidia involucrata*, 11. *Euptelea pleiosperma*, 12. *Eurya nitida*, 13. *Ilex pernyi*, 14. *Lindera erythrocarpa*, 15. *Lindera supracostata*, 16. *Litsea veitchiana*, 17. *Philadelphus subcanus*, 18. *Padus grayana*, 19. *Padus obtusata*, 20. *Padus wilsonii*, 21. *Quercus aliena*, 22. *Rhododendron huianum*, 23. *Rhododendron ririei*, 24. *Rhododendron simsii*, 25. *Stachyurus chinensis*, 26. *Tetracentron sinense*, 27. *Viburnum erubescens*.

Table S3 Niche overlapping Pianka index in FP community

	1	2	3	4	5	6	7	8	9	10	11	12	13	14
2	0.00													
3	0.21	0.30												
4	0.71	0.00	0.00											
5	0.63	0.00	0.27	0.45										
6	0.00	0.00	0.00	0.00	0.00									
7	0.20	0.00	0.52	0.00	0.39	0.00								
8	0.41	0.00	0.70	0.00	0.26	0.00	0.33							
9	0.00	0.00	0.00	0.00	0.00	0.71	0.00	0.00						
10	0.41	0.00	0.17	0.58	0.52	0.00	0.17	0.00	0.00					
11	0.00	0.00	0.08	0.00	0.35	0.18	0.07	0.15	0.26	0.45				
12	0.00	0.00	0.00	0.00	0.00	0.00	0.00	0.00	0.00	0.00	0.00			
13	0.00	0.00	0.30	0.00	0.00	0.00	0.29	0.00	0.00	0.00	0.00	0.00		
14	0.29	0.33	0.57	0.17	0.59	0.41	0.41	0.29	0.25	0.43	0.53	0.33	0.25	
15	0.00	0.00	0.16	0.00	0.12	0.00	0.31	0.00	0.00	0.31	0.41	0.00	0.00	0.33

Note: 1. *Abies chensiensis*, 2. *Abies ernestii*, 3. *Acer ceriferum*, 4. *Acer pauciflorum*, 5. *Acer franchetii*, 6. *Acer truncatum*, 7. *Carpinus cordata*, 8. *Cercidiphyllum japonicum*, 9. *Cornus controversa*, 10. *Cornus kousa*, 11. *Euptelea pleiosperma*, 12. *Lindera obtusiloba*, 13. *Quercus aliena*, 14. *Tetracentron sinense*, 15. *Tsuga chinensis*.

Table S4 Niche overlapping Pianka index in LGS community

	1	2	3	4	5	6	7	8	9	10	11	12	13	14	15	16	17	18	19	20	21	22	23	24	25	26	27	28	29	30	31	32
2	0.00																															
3	0.00	0.00																														
4	0.00	0.00	0.00																													
5	0.00	0.00	0.00	0.00																												
6	0.00	0.30	0.00	0.00	0.00																											
7	0.00	0.00	0.00	0.00	0.00	0.00																										
8	0.00	0.00	0.00	0.00	1.00	0.00	0.00																									
9	0.00	0.21	0.00	0.00	0.00	0.71	0.71	0.00																								
10	0.00	0.00	0.50	0.00	0.00	0.00	0.00	0.00	0.00																							
11	0.00	0.30	0.00	0.00	0.00	0.00	0.00	0.00	0.00	0.00																						
12	0.00	0.30	0.00	0.00	0.00	0.00	0.00	0.00	0.00	0.00	1.00																					
13	0.58	0.00	0.29	0.58	0.00	0.00	0.00	0.00	0.00	0.00	0.00	0.00																				
14	0.00	0.00	0.00	0.00	0.00	0.00	0.00	0.00	0.00	0.00	0.00	0.00	0.00																			
15	0.58	0.00	0.00	0.58	0.00	0.00	0.00	0.00	0.00	0.00	0.00	0.00	0.67	0.00																		
16	0.00	0.00	0.50	0.00	0.00	0.00	0.00	0.00	0.00	0.00	0.00	0.00	0.00	0.00	0.00																	
17	0.00	0.00	0.50	0.00	0.00	0.00	0.00	0.00	0.00	0.00	0.00	0.00	0.00	0.00	0.00	0.00																
18	0.00	0.00	0.50	0.00	0.00	0.00	0.00	0.00	0.00	0.00	0.00	0.00	0.58	0.00	0.00	0.00	0.00															
19	0.00	0.21	0.00	0.00	0.00	0.00	0.71	0.00	0.50	0.00	0.71	0.71	0.00	0.00	0.00	0.00	0.00	0.00														
20	0.00	0.00	0.00	0.00	0.00	0.00	0.00	0.00	0.00	0.00	0.00	0.00	0.00	0.00	0.00	0.00	0.00	0.00	0.00													
21	0.00	0.00	0.50	0.00	0.00	0.00	0.00	0.00	0.00	0.00	0.00	0.00	0.00	0.00	0.00	1.00	0.00	0.00	0.00	0.00												
22	0.00	0.00	0.50	0.00	0.00	0.00	0.00	0.00	0.00	0.00	0.00	0.00	0.58	0.00	0.00	0.00	0.00	1.00	0.00	0.00	0.00											
23	0.00	0.00	0.50	0.00	0.00	0.00	0.00	0.00	0.00	0.00	0.00	0.00	0.00	0.00	0.00	1.00	0.00	0.00	0.00	0.00	1.00	0.00										
24	0.33	0.70	0.17	0.00	0.00	0.33	0.00	0.00	0.24	0.00	0.00	0.00	0.19	0.33	0.19	0.33	0.00	0.00	0.00	0.33	0.33	0.00	0.33									

25	0.00	0.00	0.00	0.00	0.00	0.00	0.00	0.00	0.00	0.00	0.00	0.00	0.00	1.00	0.00	0.00	0.00	0.00	0.00	0.00	0.00	0.00	0.00	0.33								
26	0.00	0.90	0.00	0.00	0.00	0.00	0.00	0.00	0.00	0.00	0.00	0.00	0.00	0.00	0.00	0.00	0.00	0.00	0.00	0.00	0.00	0.00	0.00	0.67	0.00							
27	0.00	0.17	0.29	0.00	0.58	0.58	0.00	0.58	0.41	0.00	0.00	0.00	0.00	0.00	0.00	0.00	0.58	0.00	0.00	0.00	0.00	0.00	0.00	0.19	0.00	0.00						
28	0.00	0.00	0.00	0.00	1.00	0.00	0.00	1.00	0.00	0.00	0.00	0.00	0.00	0.00	0.00	0.00	0.00	0.00	0.00	0.00	0.00	0.00	0.00	0.00	0.00	0.00	0.58					
29	0.00	0.09	0.00	0.00	0.00	0.00	0.00	0.00	0.00	0.00	0.00	0.00	0.00	0.00	0.00	0.00	0.00	0.00	0.00	0.00	0.00	0.00	0.00	0.67	0.00	1.00	0.00	0.00				
30	0.00	0.00	0.00	0.00	0.00	0.00	0.00	0.00	0.00	0.00	0.00	0.00	0.00	0.00	0.00	0.00	0.00	0.00	0.00	1.00	0.00	0.00	0.00	0.33	0.00	0.00	0.00	0.00	0.00			
31	0.32	0.10	0.47	0.32	0.00	0.00	0.00	0.00	0.00	0.00	0.32	0.32	0.73	0.32	0.37	0.00	0.32	0.63	0.22	0.32	0.00	0.63	0.00	0.32	0.32	0.00	0.18	0.00	0.00	0.32		
32	0.34	0.29	0.43	0.34	0.24	0.29	0.24	0.24	0.38	0.19	0.24	0.24	0.50	0.19	0.50	0.14	0.34	0.19	0.34	0.14	0.14	0.19	0.14	0.47	0.19	0.14	0.50	0.24	0.14	0.14	0.63	
33	0.00	0.64	0.35	0.00	0.00	0.00	0.00	0.00	0.00	0.71	0.00	0.00	0.00	0.00	0.00	0.00	0.00	0.00	0.00	0.00	0.00	0.00	0.00	0.47	0.00	0.71	0.00	0.00	0.71	0.00	0.00	0.24

Note: 1. *Acer amplum*, 2. *Acer erianthum*, 3. *Acer flabellatum*, 4. *Betula platyphylla*, 5. *Carpinus omeiensis*, 6. *Carpinus turczaninowii*, 7. *Castanea kwetchowensis*, 8. *Castanopsis tibetana*, 9. *Clethra barbinervis*, 10. *Diospyros morrisiana*, 11. *Euonymus laxiflorus*, 12. *Eurya japonica*, 13. *Eurya nitida*, 14. *Fagus longipetiolata*, 15. *Fraxinus chinensis*, 16. *Acer maximowiczii*, 17. *Hydrangea paniculata*, 18. *Lithocarpus brevicaudatus*, 19. *Lithocarpus hancei*, 20. *Litsea elongata*, 21. *Morus australis*, 22. *Nyssa sinensis*, 23. *Oyama sinensis* , 24. *Prunus conradinae*, 25. *Cyclobalanopsis glauca*, 26. *Cyclobalanopsis myrsinifolia*, 27. *Rhododendron leigongshanense*, 28. *Schima superba*, 29. *Sorbus caloneura*, 30. *Sorbus megalocarpa*, 31. *Styrax japonicus*, 32. *Tetracentron sinense*, 33. *Viburnum betulifolium*.
